# Diagnostic value of biochemical markers (FibroTest-FibroSURE) for the prediction of liver fibrosis in patients with non-alcoholic fatty liver disease

**DOI:** 10.1186/1471-230X-6-6

**Published:** 2006-02-14

**Authors:** Vlad Ratziu, Julien Massard, Frederic Charlotte, Djamila Messous, Françoise Imbert-Bismut, Luninita Bonyhay, Mohamed Tahiri, Mona Munteanu, Dominique Thabut, Jean François Cadranel, Brigitte Le Bail, Victor de Ledinghen, Thierry Poynard

**Affiliations:** 1 Hepato-Gastroenterology, AP-HP Groupe Hospitalier Pitié-Salpêtrière, Paris, France; 2 Pathology, AP-HP Groupe Hospitalier Pitié-Salpêtrière, Paris, France; 3 Biochemistry, AP-HP Groupe Hospitalier Pitié-Salpêtrière, Paris, France; 4 Biopredictive, Paris, France; 5 Hepato-Gastroenterology, Hôpital Creil, Creil, France; 6 Pathology, Hôpital Haut Lévèque, Bordeaux, France; 7 Hepato-Gastroenterology, Hôpital Haut Lévèque Bordeaux, France; 8 Members of the LIDO and of the CYTOL Study Groups are listed at the end of the manuscript

## Abstract

**Background:**

Liver biopsy is considered as the gold standard for assessing non-alcoholic fatty liver disease (NAFLD) histologic lesions. The aim of this study was to determine the diagnostic utility of non-invasive markers of fibrosis, validated in chronic viral hepatitis and alcoholic liver disease (FibroTest, FT), in patients with NAFLD.

**Methods:**

170 patients with suspected NAFLD were prospectively included in a reference center (Group 1), 97 in a multicenter study (Group 2) and 954 blood donors as controls. Fibrosis was assessed on a 5 stage histological scale validated by Kleiner et al from F0 = none, F1 = perisinusoidal or periportal, F2 = perisinusoidal and portal/periportal, F3 = bridging and F4 = cirrhosis. Histology and the biochemical measurements were blinded to any other characteristics. The area under the ROC curves (AUROC), sensitivity (Se), specificity (Sp), positive and negative predictive values (PPV, NPV) were assessed.

**Results:**

In both groups FT has elevated and not different AUROCs for the diagnosis of advanced fibrosis (F2F3F4): 0.86 (95%CI 0.77–0.91) versus 0.75 (95%CI 0.61–0.83; P = 0.10), and for F3F4: 0.92 (95%CI 0.83–0.96) versus 0.81 (95%CI 0.64–0.91; P = 0.12) in Group1 and Group 2 respectively. When the 2 groups were pooled together a FT cutoff of 0.30 had a 90% NPV for advanced fibrosis (Se 77%); a FT cutoff of 0.70 had a 73% PPV for advanced fibrosis (Sp 98%).

**Conclusion:**

In patients with NAFLD, FibroTest, a simple and non-invasive quantitative estimate of liver fibrosis reliably predicts advanced fibrosis.

## Background

Non-alcoholic fatty liver disease (NAFLD) represents a spectrum of conditions characterized histologically by excessive accumulation of hepatic fat in the absence of alcohol consumption. Two main histological patterns of NAFLD are described: fatty liver alone and steatohepatitis (NASH). NAFLD is an increasingly recognized cause of liver-related morbidity and mortality [1-3]. Although the majority of patients do not develop complications, 28% may develop serious liver sequelae, including end-stage liver disease and hepatocellular carcinoma [1-5]. Those at highest risk include patients with significant hepatic necro-inflammation and fibrosis [1,2,6]. Therefore liver biopsy had been recommended for confirming diagnosis and for providing prognostic information [7,8].

There are several drawbacks in using liver biopsy for this purpose. This procedure is invasive, costly, and prone to complications, some minor, such as pain, others more severe with a recorded risk of death of 0.01% [9-11]. Notably, just as is the case in other chronic liver diseases, there is considerable sampling variability (40% for fibrosis staging), and a high intra and inter-pathologist variability[12,13]. Most importantly, the number of patients at risk for NAFLD is high enough that liver biopsy is not a practical and efficient tool for identifying those at risk of advanced fibrosis. Indeed an estimated 15 to 20% of the Western European population has steatosis [14] while more than half of Americans are overweight or obese.

Because liver biopsy is impossible to perform in such large cohorts of individuals, some investigators have tried to identify simple non-invasive markers of liver injury, in particular fibrosis, in patients with NASH. Different studies have shown that an age of 45 years or more, the extent of obesity, type 2 diabetes, high levels of alanine aminotransferase and triglycerides, high HOMA indices of insulin resistance, systemic hypertension, and high level of C-peptide [6,15,16] are associated with advanced fibrosis in patients with NASH. However, these findings are not consistent between studies and have been generated through retrospective studies, all amenable to biases known and unknown. Imaging techniques have moderate predictive values for advanced steatosis but not for bridging fibrosis [17,18].

In the last 3 years, we developed 2 panels of simple biochemical markers, named FibroTest (FT) and Actitest, (Biopredictive Paris, France, FibroSURE in the US patented artificial intelligence algorithm USPTO 6,631,330 ). The fibrosis index includes α_2_-macroglobulin (A2M), apolipoprotein A1, haptoglobin, total bilirubin, and γ-glutamyl-transpeptidase (GGT), and the necrotico-inflammatory activity index combines the same 5 markers, plus ALT. These panels demonstrated high predictive values for significant lesions in patients with chronic hepatitis C [19-28], chronic hepatitis B [25,29] and alcoholic liver disease [30]. These diagnostic values were also confirmed by independent groups and versus glycomics and elastometry [31-34]. Because fibrosis features are similar between patients with alcoholic liver disease and NAFLD, it was logical to hypothesize that the diagnostic value of FT would be the same.

The objective of the current study was to validate the diagnostic utility of FT for the detection of advanced fibrosis in patients with NAFLD in two prospective validation groups, one in a single center (Group 1) and one in multicenter study (Group 2). The specificity has been also assessed in a large prospective cohort of blood donors.

## Methods

### Study population

#### Group 1

The inclusion criteria were patients with suspicion of NAFLD hospitalized in our department having undergone liver biopsy. To be included patients needed to have either abnormal serum transaminases or GGT, or steatosis at ultrasonography, or one feature of the metabolic syndrome: (1) fasting glucose greater than 6.1 mmol/l or a previous diagnosis of diabetes; (2) body mass index of 27 or higher or waist circumference greater than 102 cm in men and 88 cm in women; (3) blood pressure greater than 130/85 or pharmacologically treated; (4) triglyceride-levels greater than 150 mg/dl or current use of fibrates; (5) HDL-cholesterol lower than 40 mg/dl (men) and 50 mg/dl (women). Exclusion criteria included daily alcohol consumption of at least 50 gm of pure ethanol equivalent for male and 30 gm for female during the preceding year, concomitant liver diseases (presence of HCV antibody or HBs antigen, auto-immune hepatitis, hemochromatosis diagnosed by genetic markers, Wilson's disease, alpha anti-trypsin deficiency), HIV antibody and immunosuppression, and an interval greater than 3 months between serum sample and liver biopsy. Between January 2001 and December 2004, 232 patients were hospitalized for suspicion of NAFLD; 170 patients were included and 62 patients were excluded: associated liver disease in two, missing data in 39 (FT not performed in 37 patients, biopsy not performed in 2 patients), and interval between biopsy and markers greater than 3 months in 21 patients. Characteristics are given in Table 1.

#### Group 2

These patients were patients of a prospective multicenter study (CYTOL study group). The aim of the CYTOL study was to assess the cause of chronic abnormal ALT or GGT values in patients without heavy alcohol consumption, without markers of HCV (HCV antibody), HBV (HBs antigen), autoimmune hepatitis (negative for anti-actin, anti-nuclear, anti-LKM1 antibodies), hemochromatosis (genetic markers), Wilson's disease, and alpha anti-trypsin deficiency. For the present study only the CYTOL patients with hepatic steatosis at biopsy suspected of NAFLD, were considered for inclusion. Between February 2002 and August 2004, among the 274 patients of the CYTOL study, 166 patients with steatosis at biopsy were considered for inclusion, 97 patients were included and 69 patients were excluded: 31 because followed in the training center, and 58 because the presence of miscellaneous associated liver diseases. Characteristics are given in Table 1.

### Control group

A total of 954 blood donors prospectively included were used as controls.

All patients and controls gave informed consent for use of data and serum for research purposes.

### Histological analysis

Liver biopsies were fixed, paraffin-embedded, and stained with at least hematoxylin-eosin-safran, iron staining, and Masson's trichrome or picrosirius red for collagen. A single pathologist unaware of patient characteristics analyzed the histological features (FC) in Group 1 and in Group 2 (BLB). A scoring system recently published by Kleiner et al [35] who studied inter observer variability was used. Fibrosis was staged as follows: stage 0: no fibrosis; stage 1: perisinusoidal or periportal fibrosis with 3 different patterns: 1A: mild, zone 3, perisinusoidal; 1B: moderate, zone 3, perisinusoidal fibrosis, and 1C portal/periportal fibrosis; stage 2: perisinusoidal and portal/periportal fibrosis; stage 3: bridging fibrosis; stage 4: cirrhosis. Steatosis was scored from 0 to 3 with a four grades scoring system from S0 to S3: S0_no steatosis or less than 5% (low to medium -power evaluation of parenchymal involvement by steatosis), S1_5%–33%, S2_>33%–66%, S3_>66%. Steatohepatitis was defined as a NASH score (NAS) greater than 5. The histological NAS score is defined as the unweighted sum of the scores for steatosis (0–3), lobular inflammation (0–3), and ballooning (0–2); thus ranging from 0 to 8. Cases with NAS of 0 to 2 were largely considered not diagnostic of NASH; on the other hand, most cases with scores of 5 or greater were diagnosed as NASH. Cases with activity scores of 3 and 4 were considered as borderline (probable) NASH [35].

### Serum biochemical markers

The two panel markers for the prediction of activity and fibrosis were the same as those validated in patients with chronic hepatitis C [28], B [25,29] and alcoholic liver disease [30]: 1) FibroTest (FT) includes total bilirubin, GGT, α_2_-macroglobulin, apolipoprotein A1, and haptoglobin, corrected for age and gender and is designed for a quantitative assessment of fibrosis; and 2) Actitest which includes ALT in addition to the above specified markers and is designed for a quantitative assessment of histological activity in chronic viral hepatitis. Values of FibroTest and Actitest range from zero to 1.00 with higher values indicating a greater probability of significant lesions.

AST, ALT, GGT, cholesterol, triglycerides, uric acid and total bilirubin were measured by autoanalyzer Hitachi 917 Automate (Mannheim, Germany) and Roche Diagnostics reagents (Mannheim, Germany). α_2_-macroglobulin, apolipoprotein A1, and haptoglobin were measured using an automatic nephelometer (BNII, Dade Behring; Marburg, Germany). Insulin was measured by autoanalyzer Axsym (Abbott, Irwin Texas, USA) and C-peptide by autoanalyzer IMMULIT (DPC, Los Ageles California, USA). HDL and LDL cholesterol were measured by autoanalyzer Kone (Thermo, Vantaa, Finland). The laboratory followed the recommended and validated procedures to insure reproducibility between FT components [22,23]. All the biochemical components have been prospectively assessed and assays were performed on fresh serum.

### Statistical analyses

The primary outcome was advanced fibrosis (F2, F3 and F4). In a secondary analysis, patients were classified according to the presence of severe fibrosis or cirrhosis (F3F4). Sensitivity analysis compared patients without or with moderate alcohol consumption, patients with elevated or not baseline ALT, patients without high risk of FibroTest failures, patients with baseline biopsy length smaller than 25 mm or greater, with or without fragmented biopsy and patients with paired biopsies.

The cause of discordance between presence of advanced fibrosis predicted by biochemical markers and biopsy (first biopsy if two) was attributed according to respective risk factors of failure as previously detailed [27]. Risk factors of FT failure were hemolysis, Gilbert's disease, acute inflammation and extrahepatic cholestasis. Risk factors of biopsy failure were biopsy size (less than 25 mm) and fragmentation (more than one fragment). Failure attributable to biopsy (false negative) was suspected when the biopsy was smaller than 15 mm and fragmented and without risk failure of FT [27].

Statistical analysis used Fisher's exact test, the chi-square test, Student's t test, the Mann-Whitney test and variance analysis using the Bonferroni all-pair wise and Tukey-Kramer multiple-comparison tests to take into account the multiple comparisons and multiple logistic regression for multivariate analysis. The diagnostic values of the markers were assessed using sensitivities, specificities, positive (PPV) and negative predictive values (NPV), and the areas under the receiver operating characteristic curves (AUROC). AUROC curves were calculated including FT quantitative values using empirical non-parametric method according to Delong et al [36] and compared using the method of Zhou et al [37]. For all analyses, two-sided statistical tests were used; a P-value of 0.05 or less was considered significant. Number Cruncher Statistical Systems 2003 software (NCSS, Kaysville, Utah, USA) was used for all analyses.

## Results

### Patients

A total of 170 patients were included in Group 1 and 97 in Group 2. Characteristics of patients included in Group 1 and Group 2 as well as those of the non-included groups were similar (Table 1). The only significant differences observed between Group 1 and Group 2 was for Group 2 younger mean age, less metabolic factors, more fibrosis stage 1, more severe steatosis and more NASH than in Group 1. The biopsy size in Group 1 was longer with more portal tracts than in Group 2. One case in Group 1 had a severe adverse event of biopsy with gallbladder perforation. The histological diagnosis was made during the surgical operation.

Among 267 included patients, 24 (9%) patients declared "at risk" daily alcohol consumption, between 30 g and 40 g for 17 males and between 20 g and 25 g for 7 females.

### Diagnosis of fibrosis

When compared to patients with no or mild fibrosis (F0-F1), those with advanced fibrosis (F2-F4) were older, without difference for gender (Table 2). Mean levels of α_2_-macroglobulin, total bilirubin and FT were higher and apoA1 lower. By multivariate analysis, age (P < 0.0001), α_2_-macroglobulin (P < 0.0001), GGT (P = 0.01), bilirubin (P = 0.02) and apoA1 (P = 0.048) were independently predictive of advanced fibrosis (model r^2^, 0.38; P < 0.0001).

**Figure 1:**
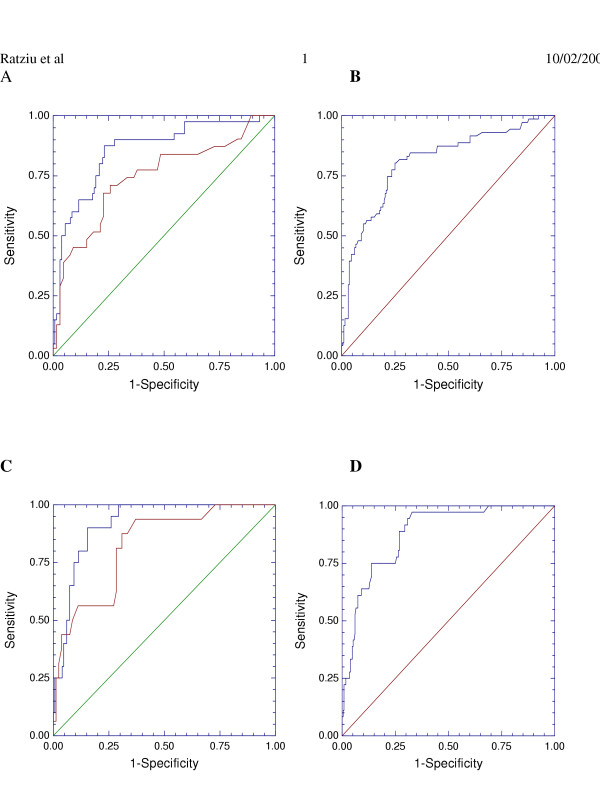
**ROC curves of the FibroTest for the detection of advanced fibrosis in Group 1 and Group 2 (A), in the overall population (B), for bridging fibrosis or cirrhosis in Group 1 and Group 2 (C), and in the overall population (D). **The diagonal line represents that achieved by chance alone (area under the curve 0.50); the ideal area under the curve is 1.00. Upper curve is Group1, lower curve is Group 2. There was no difference between the area under the ROC curves (AUROCs) for advanced fibrosis AUROC = 0.86 (95%CI 0.77–0.91) versus 0.75 (95%CI 0.61–0.83; P = 0.10), for bridging fibrosis or cirrhosis 0.92 (95%CI 0.83–0.96) versus 0.81 (95%CI 0.64–0.91; P = 0.12) in Group1 and Group 2 respectively. In the overall population the AUROCs for advanced fibrosis= 0.81 (95%CI 0.74–0.86) and for bridging fibrosis or cirrhosis = 0.88 (95%CI 0.82–0.92).

ROC curves of FT for predicting fibrosis are illustrated in Figure 1. For the primary outcome, the detection of F2 to F4 fibrosis, the area under the ROC curve was 0.86 ± 0.04. For the detection of F3-F4, the area under the ROC curve was 0.92 ± 0.04.

**Figure 2:**
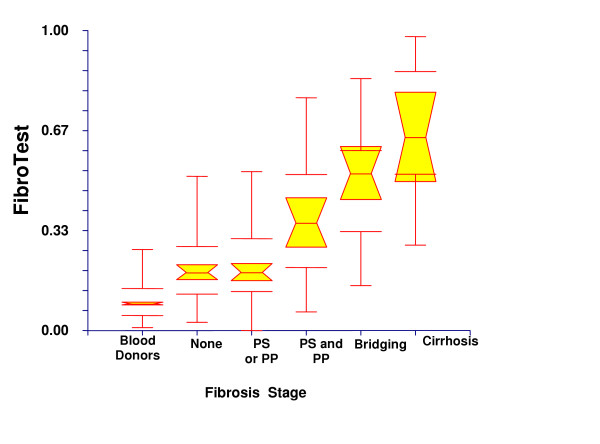
**Relationship between fibrosis stage and the FibroTest. **Notched box plots showing the relationship between the stage of fibrosis and FibroTest. The horizontal line inside each box represents the median and the width of each box the median ± 1.57 interquartile range/vn to assess 95% level of significance between group medians. Failure of the shaded boxes to overlap signifies statistical significance (*P *<0.05). The horizontal lines above and below each box encompass the interquartile range (from 25^th ^to 75^th ^percentile), and the vertical lines from the ends of the box encompass the adjacent values (upper: 75^th ^percentile plus 1.5 times interquartile range, lower 25^th ^percentile minus 1.5 times interquartile range). PS means perisinusoidal fibrosis; PP means periportal fibrosis. Spearman correlation coefficient between FibroTest and stage of fibrosis after exclusion of blood donors was = 0.44 (n = 267, P < 0.0001) and was = 0.47 (n = 1304, P < 0.0001) if blood donors were included in the analysis as subjects without fibrosis.

The distribution of FT according to the stage of fibrosis is illustrated in Figure 2. In 954 blood donors the median FT value was 0.085 (95% CI: 0.082–0.090). The median in 77 patients without fibrosis (F0) was 0.18 (0.14–0.21); in 53 patients with mild fibrosis (F1), 0.23 (0.16–0.26); in 20 patients with moderate fibrosis (F2), 0.36 (0.28–0.60); in 11 patients with bridging fibrosis (F3), 0.58 (0.45–0.80); and in 9 patients with cirrhosis (F4), 0.63 (0.52–0.91). All differences were significant (P < 0.05) except between F0 and F1 and between F3 and F4. Among blood donors the security algorithms permitted to exclude 29 (3%) patients with high risk of false positive or negative. Among the remaining 925 controls 24 (2.6%) patients had FT between 0.30 and 0.48 and none above.

Diagnostic values of FT for predicting Fibrosis in the two groups are given in Table 3 and for the overall population in Table 4. An FT score of 0.30 had 77% sensitivity and 90% negative predictive value for advanced fibrosis. An FT score of 0.70 had 98% specificity and 76% positive predictive value. FT was highly sensitive for the detection of bridging fibrosis or cirrhosis (F3F4): an FT equal to or higher than 0.30, had 92% sensitivity and 98% negative predictive value for F3F4.

### Analysis of discordance

In group 1, There was a clinically significant (2 stages or more) discordance in 17 patients (10%): 5% due to FT failure (9/170), 4% due to biopsy failure (6/170) and indeterminate in 1% (2/170). For 7 out of these 17 patients, the fibrosis stage estimated by biopsy was higher than the one estimated by FT. In one case the cause of failure was certainly attributable to FT since a well known cause of false negative was present: a case with an acute inflammation (urinary sepsis) and high haptoglobin. There was a possible cause of FT false negative in three cases with highly elevated ApoA1. In three cases there was a possible cause of failure of biopsy (poor quality: 7 mm with 3 fragments, 13 mm with 2 fragments and 21 mm with 3 fragments, respectively) with no known cause of false negative of FT. For the remaining 10 patients the fibrosis stage was two stages lower than estimated by FT. In four cases there was a certain cause of failure of FT namely Gilbert syndrome. In four cases there was a possible false negative for liver biopsy in the absence of a cause of false positive FT values: a case (25 mm, 2 fragments) with a low platelet count of 130,000/mm^3 ^and gastric varices at endoscopy; a case with a biopsy of 20 mm (1 fragment) and a low platelet count of 143,000/mm^3^; a case with a biopsy of 15 mm (5 fragments) and a low platelet count of 146,000/mm^3^; another with a biopsy of 25 mm and 2 fragments. Two cases had no attributable cause of failure: a good quality biopsy (20 mm, one fragment for one and 30 mm, one fragment for the other) with no risk factors for false positive FT values.

In group 2 there was a clinically significant (2 stages or more) discordance in 10 patients (10%): 2% due to FT failure (2/97), 2% due to biopsy failure (2/97) and indeterminate in 6% (6/97). For 9 out of these 10 patients, the fibrosis stage estimated by biopsy was higher than the one estimated by FT. In two cases the cause of failure was attributable to FT with inflammation (CRP elevated) and high haptoglobin. In one case there was a possible cause of failure of biopsy, a possible false positive with poor quality biopsy 10 mm with 3 fragments and no known cause of false negative of FT. In one case the fibrosis stage estimated by FT was higher than the one estimated by biopsy. The biopsy was small 10 mm and platelet count was low (120.000), without cause of false positive FT and therefore a false negative of the biopsy is possible.

### Diagnosis of non alcoholic steatohepatitis

FT had no significant diagnostic value for NASH (AUROC = 0.59 se = 0.06; p = 0.15). ActiTest had a statistically significant (AUROC = 0.62 se = 0.06; p = 0.04) but very weak diagnostic value for NASH.

All patients included with histological diagnosis of NASH (borderline or NASH) had steatosis. A total of 75 patients (44%) had steatosis without NASH (borderline or NASH) in group 1 and 29 (30%) in group 2. Advanced fibrosis (F2F3F4) was more frequent in patients with NASH than in those with steatosis alone: 30/95 (32%) and 30/68 (44%) in patients with NASH, versus 10/75 (13%; P = 0.005) and 1/29 (3%;P < 0.0001) in patients with steatosis alone, in group 1 and 2 respectively.

### Sensitivity analyses

Sensitivity analyses revealed that the FT AUROCs for the diagnosis of advanced fibrosis were not affected by ALT values (Table 5). ALT was not significantly higher in patients with advanced fibrosis than in those with early fibrosis (Table 2). Seventeen patients out of 40 (43%) with advanced fibrosis had ALT lower than 50 IU/L. AUROCS were higher but not significantly in patients with a biopsy size greater than 25 mm, and after exclusion of patients with Gilbert syndrome and acute inflammation (Table 5). When the 24 patients who drank at least 20 g alcohol/day in women, and 30 g/day in men have been excluded, the FT diagnostic value of the FibroTest was even higher (not significantly) with an AUROC for F2F3F4 = 0.82 (95%CI 0.74–0.87) in the remaining 243 patients (Table 5).

### FibroTest and biopsy sampling variability

A total of 47 patients had 2 liver biopsy samples collected on the same day. Fibrosis staging was concordant in 27 (57%) and discordant in 20 (43%) out of the 47 patients. The discordance was 3 stages in one case, with bridging fibrosis in one sample (F3) and no fibrosis (F0) in the other; FT was 0.40 that is F1-F2 in the conversion system. In 2 cases the discordance was of 2 stages: in one case F4 versus F2 with a FT = 0.60 (F3); one case with F3 versus F1 with a FT = 0.61 (F3). In the remaining 17 cases the discordances were of one stage. The AUROC of FT was slightly higher but not significantly when the mean between the two biopsies stages performed in the same patient was taken as the endpoint (Table 5).

### Association between components of FT and biomarkers of metabolic syndrome (Table 6)

As expected ApoA1 was highly correlated with HDL cholesterol and total cholesterol, and negatively correlated with triglycerides. A2M was highly correlated with insulinemia and C-peptide. GGT was associated with total cholesterol and LDL-cholesterol but not with triglycerides. FT was associated with insulinemia and C-peptide. There was no correlation between FT and its components with glucose and uricemia (data not shown).

## Discussion

Mass screening for significant liver injury in patients with NAFLD will be an important medical challenge in the years to come because of the epidemics of obesity and diabetes. The inability of liver biopsy to meet this challenge makes the development of non-invasive, readily available, and easy to perform serum markers, a high priority. This study highlights the potential utility of FT for the prediction of fibrosis in patients with NAFLD, as previously observed for patients infected with HCV, HBV and for patients with alcoholic liver disease.

The first validation group included patients of a secondary care center, which makes it liable to referral selection bias but the second validation group was most representative of less specialized centers. The demographic characteristics of our patients, including age and gender distribution, prevalence of cirrhosis, components of the metabolic syndrome, are similar to those reported by other studies from France[6,38]. We have taken less limited inclusion criteria concerning the alcohol consumption with inclusion of patients up to 40 g of alcohol per day for male and up to 25 g for female. There is no consensual limit. However when males with 30–45 g or women with 20–25 g or more per day were excluded following Guideline for diagnosis of NAFLD [39], the diagnostic value of FT was even better although not significantly (Table 4).

An important limitation of liver biopsy is its sampling variability [12]. The ideal gold standard should be not a 15 mm fragment but rather a 25 mm or sample [12]. In chronic hepatitis C 18 % of the discordant results between liver biopsy results and FT values have been attributed to liver biopsy failures (mostly because of small sample size) and 2% only to FT [27]. Histological lesions of NAFLD including perisinusoidal fibrosis were unevenly distributed throughout the liver parenchyma [12]. Discordant results of one stage or more between biopsy and FT were at high as 41%. Being a serum marker, FT has the advantage of representing a more global estimate of liver fibrosis throughout the whole liver. One case was emblematic of the weakness of even a 20 mm-long biopsy: a patient had severe fibrosis (F3) on the first biopsy, no fibrosis on the second biopsy (F0) and intermediate FT values (F1-F2). Although not significant, the AUROC for FT was higher when the mean fibrosis stage between the 2 biopsies was taken into account (Table 4). Contrary to histological staging systems which are all semi-quantitative, a serum biochemical marker provides a continuous quantitative assessment of liver fibrosis in 100% of patients without indeterminate cases [28].

Another drawback of liver biopsy is that for most practitioners it seems almost unethical for it to be performed in patients with normal serum transaminases values. Unfortunately, many patients with NAFLD or NASH have normal ALT and some of them have advanced liver fibrosis [40,41]. In the present study ALT was lower than 50 IU/L in 43% of patients with advanced fibrosis. As in chronic hepatitis C, FT AUROCs for the diagnosis of advanced fibrosis in NAFLD were unchanged in patients with ALT values lower than 50 IU/L (Table 4); Therefore FT could allow the diagnosis of fibrosis even in patients that are not eligible for liver biopsy.

A few other markers have been evaluated in NAFLD for the diagnosis of fibrosis. Sakugawa et al, demonstrated in 112 patients with NAFLD, good diagnostic values for hyaluronic acid and type IV collagen 7S for stage F3 and F4 with AUROCs of 0.80 and 0.83 respectively [42]. Lainé et al combined hyaluronic acid and the carbohydrate-deficient transferrin/transferrin ratio in 173 patients with increased serum aminotransferases and features of metabolic syndrome [38]. Hyaluronic acid AUROC for F2F3F4 fibrosis was 0.92. In our experience in patients with chronic hepatitis C and alcoholic liver disease FT had a higher sensitivity than hylauronic acid, especially for the diagnosis of moderate fibrosis [20,30]. Rosenberg et al. studied a panel combining age, hyaluronic acid, amino terminal propeptide of type III collagen and tissue inhibitor of matrix metaloprotein-1 in 81 patients with NAFLD [43]. They observed an AUROC of 0.87 for advanced fibrosis, similar to FT AUROC in the present study [43].

When comparing the performance of different serum markers for liver fibrosis the diagnostic yield is far from being the only aspect that needs to be considered [44]. Equally important are the description of analytical conditions for serum measurements including intra patient and intra sample variability, the description of precautions of use and the identification of cases with discordant results between serum markers and liver biopsy as well as risk factors for these discordances [22,23,27,45]. In the present study we observed 5% of discordances due to FT failure versus 4% due to biopsy failure. We recognize that in the analysis of discordance, we assert that in patients with no known cause of false negative FT, and a small length biopsy, we consider because of biopsy sampling error that the discordance was due to failure of biopsy. We acknowledge that in these cases there is no direct prove of failure of biopsy when no second biopsy and no other independent marker have been performed. As previously described Gilbert's syndrome and acute inflammation were the most frequent causes of FT failures. We observed a possible cause of false negative FT failure, not previously described: an unusual high serum apoA1 concentration due to high serum HDL-cholesterol (correlation between ApoA1 and HDL cholesterol R = 0.77). This condition was rare (3 cases out of 170, 1.8%) but is probably more frequent than in other chronic liver diseases without lipids abnormalities.

None of the FT components is a direct marker of hepatic extracellular matrix nevertheless the overall score is correlated with liver fibrosis. A2M is a protease inhibitor, but also has multiple functions as a binding, carrier and targeting protein [46]. A2M is associated with several growth factors: fibroblast, vascular endothelial, epidermal, transforming and platelet derived growth factors [19]. Interestingly, in patients with NAFLD, the present study demonstrated a very significant association between A2M and insulin levels, a hallmark of insulin resistance. Relationships between A2M and insulin have been described for more than 40 years [47]. Some studies have observed an increase of A2M in diabetic patients [48]. Insulin is covalently bound to A2M in plasma [49] and A2M is a binding protein of Insulin-like Growth Factor Binding Protein-1 (IGFBP-1) which modifies the IGFBP-1/IGF interaction [50]. Therefore A2M can be directly involved both in the hepatic mechanisms of insulin resistance and fibrogenesis [50,51].

When compared to alcoholic liver disease the decrease of apolipoprotein A1 was not significant in patients with advanced fibrosis and NAFLD. The interpretation of this negative observation must be prudent because of the small number of patients with cirrhosis included in our NAFLD population. We observed a dramatic decrease in ApoA1 serum levels in patients with alcoholic liver disease which was associated with necrosis, polymorphonuclear infiltrate and Mallory bodies [30]. In the present NAFLD population the prevalence of patients with severe necrosis was small, and only 2 patients had a polymorphonuclear infiltrate (1.2%). In the first case there was a dramatic decrease in ApoA1 (0.05 g/L), as observed in alcoholic steato-hepatitis. In the second case the absolute value of ApoA1 was not decreased (1.72 g/L) but was relatively low in comparison with HDL cholesterol (1.64 mmol/L).

Although in NAFLD there is no specific treatment approved to treat liver injury, the diagnosis of advanced fibrosis could be very important to motivate the patient for diet or lifestyle modifications, for intensive treatment of complications of the metabolic syndrome or for weighing in favour of anti-obesity surgery. The early detection of advanced fibrosis is the first step to reduce future cirrhosis-related deaths. Diagnosing silent cirrhosis has important consequences in terms of screening for portal hypertension and hepatocellular carcinoma, of preventing complications and of timely indication for liver transplantation.

Based on our data, a preliminary algorithm for the use of FT as a screening tool in patients at risk for NAFLD can be suggested. Below 0.30 the probability of cirrhosis is very low and there is no need for ultrasonography or endoscopy. Between 0.30 and 0.70 it is mandatory to help the patient in reducing all metabolic factors (overweight, diabetes, dyslipidemia and maybe complete alcohol abstinence). Follow-up of these patients with FT can be advised. When the FT value is 0.70 or higher, the patient should be managed as a patient with cirrhosis, and surveillance by ultrasonography and endoscopy should be implemented in order to prevent potentially severe complications of cirrhosis.

## Conclusion

In patients with NAFLD, FibroTest-FibroSURE, a simple and non-invasive quantitative estimate of liver fibrosis reliably predicts advanced fibrosis

## Abbreviations

A2M alpha2macroglobulin; GGT, γ-glutamyl-transpeptidase; ALT, alanine aminotransferase; ROC, receiver operating characteristic; AST, aspartate aminotransferase; NPV, negative predictive value; PPV, positive predictive value; ULN, upper limit of normal.

## Competing interests

TP is a consultant and has a capital interest in Biopredictive, the company marketing FibroTest-Actitest.

## Authors' contributions

VR and TP conceived and wrote the manuscript. VR, TP, LB, MM, DT, JFC, and VDL were responsible for the patient drafting, and participated in the coordination of the study. DM and FIB drafted the paper. FC and BLB were responsible of histological analysis. TP performed the statistical analysis. All authors read and approved the final manuscript

### Members of the LIDO (Liver Injury in Diabetes and Obesity) study group are

André Grimaldi, Philippe Giral, Eric Bruckert, Gérard Turpin, Agnès Heurtier, Sophie Gombert, Francine Lamaison, Joseph Moussalli, Sophie Le Calvez, Yves Benhamou, Cecilia D'Arrondel, Arnaud Cocaul, Isabelle Ravalet, Stéphanie Combet, Hôpital Pitié Salpêtrière; Philippe Podevin, Hôpital Cochin; Arnaud Basdevant, Gérard Slama, Karine Clement, Hôpital Hotel-Dieu; Lawrence Serfaty, Chantal Housset, Jacqueline Capeau, Hôpital Saint Antoine.

### Members of the CYTOL study group are

Alain Blanchi, Christophe Pilette Hôpital du Mans, Marc Bourlière, Valérie Oulès, Hôpital St Joseph Marseille, Christophe Renou Hôpital d'Hyères, Dominique Capron Hôpital d'Amiens, Frédéric Oberti, Paul Calès Hôpital d'Angers, Albert Tran, Eve Gelsi Hôpital de Nice, Jérôme Gournay Hôpital de Nantes, Anais Vallet-Pichard, Stanislas Pol Hôpital Necker, Paris, Xavier Causse Hôpital d'Orléans.

## Pre-publication history

The pre-publication history for this paper can be accessed here:



## Figures and Tables

**Table 1:** Characteristics of the patients included or non included in Group 1 and Group 2

	**Group 1**			**Group 2**			
**Characteristic**	**Included** ** N = 170**	**Non-included** ** N = 62**	**Significance p**	**Included N = 97**	**Non-included** ** N = 69**	**Significance p**	**Significance p training** ** vs validation**

**Demographics**							
Age at biopsy, *mean (se)*, *years*	52.8 (0.87)	54.9 (1.47)	0.18	48.5 (1.28)	49.1 (1.45)	0.18	0.01
Male, *n (%)*	98 (58)	39 (63)		57 (59)	38 (69)	0.64	0.86
**Metabolic factor (%)**							
Diabetes	61/170 (36)	10/62 (16)	0.004	31/97 (32)	8/97 (12)	0.62	0.001
Arterial Hypertension	53/170 (31)	6/62 (10)	0.14	15/97 (16)	16/97 (23)	0.21	0.001
BMI greater than 27 kg/m^2^	85/141 (60)	12/21 (57)	0.78	43/97 (44)	22/97 (32)	0.11	0.42
Triglycerides greater than 1.5	68/161 (42)	17/42 (40)	0.87	35/97 (36)	15/97 (22)	0.047	0.33
HDL cholesterol lower than 40 mg/L	36/151 (24)	6/38 (16)	0.29	15/94 (16)	15/66 (23)	0.28	0.14
Metabolic factor							
None	21/170 (24)	34/62 (55)	P < 0.0001			P < 0.0001	
At least one	134/170 (79)	28/62 (45)					
**Alcohol consumption**, *n (%)*							
No	118/170 (69)	51/62 (79)	0.63	57/97 (59)	41/69 (59)	0.63	0.20
Yes	52/170 (31)	11/62 (21)		40/97 (41)	28/69 (41)		
**Fibrosis stage**, *n (%)*							
*Non advanced fibrosis*	*130/170 (76)*	*59/60(98)*	*0.001*	*66/97(68)*	*51/69(74)*	*0.48*	*0.13*
No fibrosis (F0)	77/170 (45)	39/60 (65)		26/97 (27)	21/69 (30)		
Perisinusoidal or periportal (F1)	54/170 (31)	20/60 (33)		40/97 (41)	30/69 (43)		
1A: mild, zone 3, perisinusoidal	29/170 (17)	19/60 (31)		18/97 (19)	18/69 (25)		
1B: moderate, zone 3, perisinusoidal	2/170 (1) 1/60 (2)			0/97 (0) 22/97 (22)	0/69 (0) 12/69 (18)		
1C: portal/periportal	22/170 (13)	0/60 (0)		*31/97 (32)*	*18/69(26)*		
*Advanced Fibrosis*	*40/170 (24)*	*1/60(2)*		15/97 (15)	13/69 (19)		
Perisinusoidal and portal/periportal (F2)	20/170 (12) 11/170 (7)	0/60 (0) 0/60 (0)		12/97 (12) 4/97 (4)	2/69 (3) 3/69 (4)		
Bridging (F3)	9/170 (5)	1/60 (2)					
Cirrhosis (F4)							
**Non alcoholo-steato-hepatitis**, *n (%)*							
No	75/170 (44)	25/60 (41)	0.15	29/97 (30)	40/69(58)	0.001	0.003
Borderline (Probable)	67/170 (39)	26/60 (44)		35/97 (36)	19/69 (28)		
Yes	28/170 (17)	9/60 (15)		33/97 (34)	10/69 (14)		
**Steatosis**							
*Histology*							
Absence or <5%	22/170 (13)	12/60 (20)	0.38	19/97 (20)	13/69 (19)	0.38	0.22
6–32	57/170 (34)	14/60 (23)		22/97 (23)	27/69 (39)		
33–65	43/170 (25)	16/60 (27)		27/97 (28)	10/69 (14)		
66–100	48/170 (28)	18/60 (30)		29/97 (30)	19/60 (28)		
**Interval between biopsy and serum,**							
*median*	0	-100	<0.001	0	0	0.90	0.07
*(95%CI),*	[0; 1]	[-215; 0]		[0; 0]	[0; 0]		
*Range days*	-87;+89	-538; +540		-24;+49	-42; +40		
**Biopsy quality ***mean (se)*							
Sample size	20.0 (0.5)	19.7 (0.9)	0.68	17.8 (0.7)	21.4 (0.9)	0.001	0.007
Number of fragment	2.3 (0.2)	2.3 (0.2)	0.41	2.3 (0.2)	2.2 (0.2)	0.76	0.52
Number of portal tracts	16.3 (0.6)	16.3 (1.1)	0.79	13.6 (0.6)	17.1 (1.0)	0.008	0.01
**Serum biochemical markers**, *mean (se)*							
α_2_-macroglobulin ^(*g*/*L*)^	1.68 (0.05)	1.78 (0.11)	0.26	1.72 (0.06)	1.76 (0.08)	0.47	0.38
ALT *(IU)*	71 (3)	64 (6)	0.26	79 (5)	84 (10)	0.64	0.23
AST *(IU)*^†^	50 (3)	42 (3)	0.10	45 (3)	52 (5)	0.10	0.009
Apolipoprotein A1(*g/L*)	1.47 (0.03)	1.46 (0.07)	0.84	1.59 (0.07)	1.53 (0.04)	0.80	0.07
GGT (*IU/L*)	110 (11)	113 (19)	0.96	132 (12)	158 (19)	0.96	0.03
Haptoglobin (*g/L*)	1.10 (0.04)	1.19 (0.15)	0.69	1.12 (0.10)	1.02 (0.05)	0.16	0.28
Total bilirubin (μ*mol/L*)	14.5 (3.1)	12.8 (2.0)	0.43	12.6 (0.6)	13.6 (1.0)	0.89	0.64
FibroTest (*0.00–1.00*)	0.30 (0.02)	0.37 (0.05)	0.18	0.29 (0.02)	0.33 (0.02)	0.21	0.84
ActiTest (*0.00–1.00*)	0.39 (0.02)	0.34 (0.04)	0.24	0.41 (0.02)	0.43 (0.03)	0.55	0.26

**Table 2:** Univariate and multivariate predictors of advanced fibrosis

Characteristic	Group 1			Group 2			Multivariate analysis	
							All patients included	
	
	F0-F1 n = 130	F2-F4 n = 40	P-value	F0-F1 N = 66	F2-F4 N = 31	P-value	Odds Ratio (95% CI) N = 267	P-value
Age years	50.4 (1.0)	60.4 (1.4)	<0.0001	5.3 (1.4)	55.4	<0.0001	1.07 (1.03–1.10)	<0.0001
Male	78 (60%)	20 (50%)	0.26	39 (59%)	18 (58%)	0.92	0.83 (0.47–1.64)	0.59
Markers α_2_-macro g/L	1.52 (0.05)	2.22 (0.13)	<0.0001	1.56 (0.06)	2.04 (0.14)	0.007	3.74 (2.2–6.5)	P < 0.0001
ALT IU/L	73 (4)	66 (7)	0.30	73 (6)	91 (10)	0.09	1.00 (0.99–1.01)	0.10
AST IU/L	46 (2)	64 (10)	0.25	36 (2)	64 (7)	<0.0001	1.03 (1.01–1.05)	0.01
ApoA1 g/L	1.48 (0.03)	1.43 (0.06)	0.99	1.63 (0.10)	1.49 (0.05)	0.26	0.32 (0.10–0.99)	0.048
Haptoglobin g/L	1.12 (0.04)	1.05 (0.10)	0.20	1.17 (0.13)	1.01 (0.10)	0.42	0.81 (0.45–1.46)	0.47
GGT IU/L	95 (10)	158 (34)	0.09	131 (14)	132 (23)	0.87	1.01 (1.01–1.01)	0.01
Bilirubin, μmol/L	10.7 (0.5)	26.9 (13.2)	0.003	12.6 (0.7)	12.5 (1.0)	0.89	1.06 (1.01–1.11	0.03
FibroTest	0.23 (0.01)	0.51 (0.03)	<0.0001	0.24 (0.02)	0.41 (0.04)	<0.0001	439 (73–2640)*	<0.0001
ActiTest	0.37 (0.02)	0.43 (0.03)	0.11	0.37 (0.02)	0.50 (0.04)	0.004	0.94 (0.19–4.73)*	0.93

All data are means (se) and proportions (n [%]). ALT, alanine aminotransferase; AST, aspartate aminotransferase; GGT, γ-glutamyl-transpeptidase.

*Only FibroTest and ActiTest values were entered in this model

**Table 3:** Diagnostic value of FibroTest for predicting Fibrosis in the two groups

	**Group 1**				**Group 2**			
**Cut-off of FibroTest**	**Sensitivity**	**Specificity**	**Positive Predictive** ** Value**	**Negative Predictive** ** Value**	**Sensitivity**	**Specificity**	**Positive Predictive** ** Value**	**Negative Predictive** ** Value**
**Stage F2F3F4**			Prevalence = 0.24 (40/170)				Prevalence = 0.32 (31/97)	

0.30	0.83 (33/40)	0.78 (101/130)	0.53 (33/62)	0.94 (101/108)	0.71 (22/31)	0.74 (49/66)	0.56 (22/39)	0.84 (49/58)
0.70	0.18 (7/40)	0.98 (128/130)	0.78 (7/9)	0.80 (128/161)	0.13 (4/31)	0.98 (65/66)	0.80 (4/5)	0.71 (65/92)
**Stage F3F4**			Prevalence = 0.12 (20/170)				Prevalence = 0.16 (16/97)	
0.30	0.95 (19/20)	0.71 (107/150)	0.31 (19/62)	0.99 (107/108)	0.88 (14/16)	0.69 (56/81)	0.36 (14/39)	0.97 (56/58)
0.70	0.25 (5/20)	0.97 (146/150)	0.56 (5/9)	0.91 (146/161)	0.25 (4/16)	0.99 (80/81)	0.80 (4/5)	0.87 (80/92)
% of patients with FT between 0.30–0.70	0.32 (54/170)				0.35 (34/97)			

**Table 4:** Diagnostic value of FibroTest for predicting Fibrosis in all patients

**Cut-off of** ** FibroTest**	**Sensitivity**	**Specificity**	**Positive Predictive** ** Value**	**Negative Predictive** ** Value**
**Stage F2F3F4**			Prevalence = 0.27	

0.30	0.77 (55/71)	0.77 (150/196)	0.54 (55/101)	0.90 (150/166)
0.70	0.15 (11/71)	0.98 (192/196)	0.73 (11/15)	0.76 (192/252)
**Stage F3F4**			Prevalence = 0.13	
0.30	0.92 (33/36)	0.71 (163/231)	0.33 (33/101)	0.98 (163/169)
0.70	0.25 (9/36)	0.97 (225/252)	0.60 (9/15)	0.89 (225/252)
% of patients with FT between 0.30–0.70	0.33 (88/267)			

**Table 5:** Sensitivity analyses of FibroTest for the diagnosis of advanced fibrosis (F2F3F4) [Area under the ROC curves (AUROCs)], according to group, ALT values, alcohol consumption, exclusion of high risk of FibroTest failures, quality of biopsy and repeated biopsies

**Characteristic of patients**	**n**	**FibroTest AUROC**	**Lower 95%**	**Upper 95%**	**Prevalence advanced** ** fibrosis F2F3F4**
**All patients**	267	0.81	0.74	0.86	0.27
**Stratified according to Group**					
Group 1	170	0.86	0.77	0.91	0.24
Group 2	97	0.75	0.61	0.83	0.32
**Stratified according to ALT value**					
ALT<50IU/L	99	0.80°	0.65	0.90	0.24
ALT>= 50 IU/L	168	0.81	0.72	0.87	0.28
**Stratified according to Alcohol consumption**					
<30 g for men and <20 g for female	243	0.82 $	0.74	0.87	0.32
30–40 g for men or 20–25 g for female	24	0.71	0.66	0.84	0.24
**High risk of FibroTest failures excluded**					
Gilbert excluded	263	0.82	0.75	0.88	0.26
Acute Inflammation excluded	263	0.82	0.75	0.87	0.26
Gilbert or Acute Inflammation excluded	258	0.83	0.76	0.88	0.26
**Quality of biopsy**					
<25 mm	202	0.80 *	0.72	0.86	0.29
>= 25 mm	65	0.85	0.66	0.94	0.20
Fragmented	152	0.81 £	0.67	0.89	0.29
Not fragmented	115	0.81	0.71	0.87	0.23
**Patients with 2 biopsies**					
First biopsy	47	0.87	0.71	0.95	0.34
Second biopsy	47	0.90	0.75	0.96	0.32
Mean 2 biopsies	47	0.91	0.76	0.97	0.32

^P = 0.10 Group 1 vs Group 2

° P = 0.72 non-elevated vs elevated transaminases ALT

$ P= 0.04 <30 g for men and <20 g for female vs 30–40 g for men or 20–25 g for female

* P = 0.47 <25 mm vs >= 25 mm

£ P = 0.96 fragmented vs not fragmented

**Table 6:** Association between biochemical markers of fibrosis and biochemical metabolic markers

**Biochemical markers**	**Total cholesterol N = 161**	**HDL Cholesterol N = 153**	**LDL Cholesterol N = 153**	**Triglycerides N = 161**	**Insulin N = 60**	**C-Peptide N = 59**
α_2_-macroglobulin	-0.10 *	0.07	-0.09	0.01	**0.41**	**0.42**
	0.23 $	0.38	0.29	0.92	**0.001**	**0.001**
Apolipoprotein A1	**0.30**	**0.77**	0.10	**-0.23**	0.02	0.00
	**<0.0001**	**<0.0001**	0.21	**0.003**	0.87	0.99
Haptoglobin	0.14	-0.04	**0.20**	0.12	-0.01	0.19
	0.07	0.61	**0.01**	0.11	0.94	0.14
GGT	0.24	0.07	**0.24**	0.12	0.12	0.25
	0.002	0.40	**0.002**	0.14	0.36	0.06
Total bilirubin	0.03	0.14	0.05	**-0.17**	-0.06	-0.09
	0.74	0.09	0.50	**0.03**	0.62	0.48
FibroTest	**-0.16**	-0.11	-0.07	0.01	**0.29**	**0.33**
	**0.04**	0.17	0.40	0.89	**0.02**	**0.01**

*Spearman coefficient of correlation

$ Significance P value

There was no significant correlation between FT and its components with glucose and uricemia (data not shown).
